# Multimodal prehabilitation in elective oncological colorectal surgery enhances preoperative physical fitness: a single center prospective real-world data analysis

**DOI:** 10.2340/1651-226X.2024.20287

**Published:** 2024-02-21

**Authors:** David W.G. ten Cate, Charissa R. Sabajo, Charlotte J.L. Molenaar, Loes Janssen, Bart C. Bongers, Gerrit D. Slooter

**Affiliations:** a Department of Surgery, Máxima Medical Center, De Run 4600, 5504 DB Veldhoven, the Netherlands; b Department of Nutrition and Movement Sciences, School of Nutrition and Translational Research in Metabolism (NUTRIM), Faculty of Health, Medicine and Life Sciences, Maastricht University, Universiteitssingel 50, 6629 ER Maastricht, the Netherlands; c Department of Surgery, School of Nutrition and Translational Research in Metabolism (NUTRIM), Faculty of Health, Medicine and Life Sciences, Maastricht University, Universiteitssingel 50, 6629 ER Maastricht, the Netherlands

**Keywords:** Preoperative fitness, prehabilitation, colorectal surgery

## Abstract

**Background:**

Surgery can lead to curation in colorectal cancer (CRC) but is associated with significant morbidity. Prehabilitation plays an important role in increasing preoperative physical fitness to reduce morbidity risk; however, data from real-world practice is scarce. This study aimed to evaluate the change in preoperative physical fitness and to evaluate which patients benefit most from prehabilitation.

**Materials and Methods:**

In this single-arm prospective cohort study, consecutive patients undergoing elective colorectal oncological surgery were offered a 3- to 4-week multimodal prehabilitation program (supervised physical exercise training, dietary consultation, protein and vitamin supplementation, smoking cessation, and psychological support). The primary outcome was the change in preoperative aerobic fitness (steep ramp test (SRT)). Secondary outcomes were the change in functional walking capacity (6-minute walk test (6MWT)), and muscle strength (one-repetition maximum (1RM) for various muscle groups). To evaluate who benefit most from prehabilitation, participants were divided in quartiles (Q1, Q2, Q3, and Q4) based on baseline performance.

**Results:**

In total, 101 patients participated (51.4% male, aged 69.7 ± 12.7 years). The preoperative change in SRT was +28.3 W, +0.36 W/kg, +16.7% (P<0.001). Patients in all quartiles improved at the group level; however, the relative improvement decreased from Q1-Q2, Q2-Q3, and Q3-Q4 (P=0.049). Change in 6MWT was +37.5 m, +7.7% (P<0.001) and 1RM improved with 5.6-33.2 kg, 16.1-32.5% for the various muscle groups (P<0.001).

**Conclusion:**

Prehabilitation in elective oncological colorectal surgery is associated with enhanced preoperative physical fitness regardless of baseline performance. Improvements were relatively larger in less fit patients.

## Background

Colorectal cancer (CRC) is the third most commonly diagnosed cancer in the Netherlands [1]. Although surgery is the cornerstone of treatment for CRC, up to 29% of patients have a complicated course following surgery [2]. The sequelae of complications may result in delayed or incomplete recovery, prolonged length of hospital stay (LoS), risk of readmission, and/or even permanent loss of physical functioning [3]. To improve surgical outcomes, it is vital to reduce the incidence and impact of postoperative complications and to accelerate postoperative recovery of physical functioning. This might also be important for long-term outcomes, such as return to normal daily activities and quality of life. A strong and independent predictor of postoperative complications after CRC surgery is preoperative physical fitness, in which lower physical fitness is associated with a higher risk for postoperative complications [4–6]. Prehabilitation programs aim to improve a patient’s physical, nutritional, metabolic, and mental health status preoperatively using multimodal interventions and, as such, these may play an important role in improving postoperative outcomes [7, 8].

Traditionally, the randomized controlled trial (RCT) has been considered the gold standard for evaluating the effectiveness of interventions. To date, several RCTs have been performed to evaluate the efficacy and safety of prehabilitation programs, in which a lower complication rate [9–11], shortened LoS [10] and improved preoperative physical fitness [9–12] have been demonstrated. A role for prehabilitation has been recognized in the most recent guideline on enhanced recovery after surgery (ERAS) [13]. However, RCTs have limited external validity and applicability to routine practice, as they merely provide evidence of efficacy but not about true effectiveness (benefit to patients in daily clinical practice). That is generally because patients and practitioners in RCTs are different from those in routine practice and elderly and comorbid patients are underrepresented [14]. Few study groups used real-world data indicating that prehabilitation may prevent postoperative complications after CRC surgery [15–18], however they mostly included frail patients. The aim of this study was to evaluate the change in preoperative physical fitness in patients scheduled for elective CRC surgery participating in a multimodal prehabilitation program that was implemented in the CRC pathway as standard of care, while also evaluating which patients benefit most from prehabilitation in terms of preoperatively improving physical fitness.

## Material and methods

### Study design and participants

In this single-arm prospective cohort study, based on the STROBE guideline, consecutive patients scheduled for elective CRC surgery between January 2020 and June 2021 at the Máxima Medical Center (MMC), a teaching hospital in Veldhoven, the Netherlands, were offered a multimodal prehabilitation as part of usual care. The program was limitedly available during the coronavirus disease (COVID-19) pandemic and during the international PREHAB trial [11]. Every patient who was willing and able to participate was included. Moreover, data of these patients needed to be availabe at baseline (directly after diagnosis, T0) and after completion of the program (prior to surgery, approximately 3–4 weeks after baseline, T1) of at least one exercise test (i.e. aerobic fitness, functional walking capacity, and/or muscle strength). Patients unable to perform physical exercise due to paraplegia or orthopedic impairments were excluded. Patients who participated in the international PREHAB trial for multimodal prehabilitation were also excluded [19].

### Multimodal prehabilitation program

The program consists of four pillars and the duration was approximately 3–4 weeks [11]. The main difference with the previously published prehabilitation protocol [19] is that patients did not perform a preoperative cardiopulmonary exercise test (CPET), as this test was not part of routine practice.

### Clinical setting

Directly after the multidisciplinary team meeting at which diagnosis and treatment were confirmed, patients were contacted to participate in the program. Time from the multidisciplinary team to surgery was approximately 4–5 weeks, creating the time period for the multimodal prehabilitation program. In case patients with rectal cancer were treated with neoadjuvant therapy, they participated in the program in the 4 weeks prior to surgery.

Patients met with the case manager for an extensive explanation about both the program and the surgical procedure. All patients were screened for anemia. In case of iron deficient anemia, patients were treated with Ferinject 1,000 mg intravenously or referred to an internal medicine physician for further investigation. Every patient selected for colorectal surgery was treated conform the ERAS protocol [13]. Minimally invasive surgery was the preferred surgical approach.

### Physical exercise training

Patients trained three times a week under supervision of a physical therapist at the hospital or at a community physical therapy practice based on their preference. All therapists were trained to supervise the program. Physical exercise training consisted of high-intensity interval training (HIIT) complemented by resistance exercises to preoperatively improve both aerobic fitness and muscle strength.

### High-intensity interval training

Each HIIT session duration was 28–32 min and included a 4-min warm-up at a moderate exercise intensity, which was followed by 4 high-intensity intervals of 2–3 min alternated with 4 moderate-intensity intervals of 4 min. The steep ramp test (SRT) was used to individualize the intensity of HIIT using the following equation [20]:

CPET WR_peak_ = (0.65 × SRT WR_peak_) – 3.88

The work rate at peak exercise of CPET (CPET WR_peak_) was estimated and the high-intensity interval intensity was set at 90% of CPET WR_peak_.

The SRT is a short and practical maximal exercise test on a cycle ergometer without respiratory gas analysis, in which the reached WR_peak_ represents its primary outcome measure [21]. The personalized training intensity aimed to result in a metabolic response ranging from 85–100% of the oxygen uptake at peak exercise, a heart rate of 85–100% of the maximal heart rate, and a 6–20 Borg score for rating of perceived exertion of 16–18 during the high-intensity intervals. Training intensity of the moderate-intensity intervals was set at 30% of CPET WR_peak_, thereby aiming for recovery at a level around or just below the patient’s ventilatory anaerobic threshold. If a patient was unable to complete the high-intensity intervals for 4 periods of at least 2 min (<8 min in the high-intensity range), training intensity was reduced by steps of 10%.

### Resistance training

After each HIIT session, patients completed a resistance training program. The program took approximately 30 min and consisted of 2 sets of 10 repetitions of the low row, chest press, leg press, and lateral pull down. Training intensity was gradually increased every week based on the indirect measurement of the exercise-specific one-repetition maximum (1RM) [22], and increased from 65% of the exercise-specific 1RM in week 1, to 70% of the 1RM in week 2, and 75% of the 1RM in week 3 and 4.

### Low-intensity physical activity

On weekdays at which no supervised training session was scheduled, patients were instructed to perform low-intensity walking or cycling for a total of 60 min per day.

### Nutritional support

At the start of the prehabilitation program, a consultation with a dietician was scheduled where standard care measurements and tests were used. During consultation, caloric and protein intake was assessed and advice on target protein intake or energy-enriched nutrition were given. Target protein intake was 1.5–1.8 g/kg body mass in all patients [23, 24] and advice was provided to spread protein intake across meals. Patients received high-quality protein supplements (Refit®-TMP-90-Shake, Friesland Campina Domo, Amersfoort, the Netherlands) following physical exercise training. Vitamin D was advised following the Dutch guideline [25]. In case of renal dysfunction (estimated glomerular filtration rate <60 ml/min), patients did not receive protein supplementation.

### Cessation of smoking

The smoking-cessation program was outsourced to Sinefuma (Sinefuma B.V., Breda, the Netherlands) [26]. Sinefuma designed a special prehabilitation cessation program with personal coaching and nicotine replacement therapy.

### Psychological support

Patients received extensive information about the prehabilitation program, surgery and postoperative care during their hospital stay, and received instructions on breathing and relaxation techniques. Moreover, the need and patient’s wish of additional consultation with a medical psychologist was discussed with the patient.

### Outcome measures

#### Aerobic fitness

Based on its high criterion validity [20, 27], the SRT was also used to assess and monitor changes in aerobic fitness. The SRT was carried out according to a standardized protocol on an advanced cycle ergometer (Lode Corival/Excalibur B.V. Groningen, the Netherlands) in upright position. The primary outcome of the SRT was the achieved WR_peak_ [27].

#### Functional walking capacity

The 6-minute walk test (6MWT) was conducted according to a standardized protocol [28]. The attained walking distance in meters was the primary outcome measure of the 6MWT.

#### Muscle strength

The 1RM was calculated for low row, chest press, leg press, and lateral pull down muscle groups using the following formula:

1RM (kg) = used load/(1.0278 – (0.0278 × number of repetitions)) [22]

### Study outcomes

The primary outcome of this study was the change in preoperative aerobic fitness during the multimodal prehabilitation program. At baseline (directly after diagnosis, T0) and after completion of the program (prior to surgery, approximately 3–4 weeks after baseline, T1) patients performed the SRT. Both the absolute (W) and relative (W/kg body mass) change in SRT WR_peak_ were analyzed. Secondary outcomes of this study were the change in 6MWT, percentage of patients with a progression on 6MWT of >20 m [29] and 1RM following the program. Patients performed the 6MWT and 1RM tests of low row, chest press, leg press, and lateral pull down at T0 and T1. For the 1RM, both the absolute (kg) and relative (kg/kg body mass) change were analyzed. Other outcomes involved training adherence (calculated as a the percentage completed trained sessions of the total number of planned training sessions), age, sex, BMI, American Society of Anesthesiologist score (ASA), Charlson comorbidity index score, pathological tumor, node and metastasis status (pTNM), surgical data, postoperative complications (independently graded by DtC and CS; discrepancies were discussed until agreement with GS), comprehensive complication index (CCI), and LoS. The CCI is a validated measurement that weighs all complications into a sum score ranging from 0 to 100 [30]. A CCI score >20, was chosen to define severe complications [11]. Serious adverse events in this study were defined and only registered in case the event would be related to participation in the prehabilitation program.

### Statistical analysis

Data analysis was performed using Statistical Package for the Social Sciences (version 22; IBM, SPSS Inc., Chicago, IL, USA). Data were analyzed through complete case analysis. Data were checked for normality using Kolmogorov–Smirnov Test and the Shapiro–Wilk Test. To assess the effect of prehabilitation on preoperative physical fitness, changes in SRT, 6MWT, and 1RM between T0 and T1 are presented as absolute, normalized for body mass (SRT and 1RM), and relative (%) change scores. Differences between T0 and T1 were tested for statistically significant differences with either paired samples *t*-tests or Wilcoxon signed rank tests, as appropriate. To evaluate which patients improved most in preoperative physical fitness, subgroup analyses were performed based on test performance at T0 (participants divided in quartiles), age groups (<49, 50–60, 61–70, 71–80, 81>), sex (independent samples *t*-test), and ASA score using analysis of variance (ANOVA) or Kruskal–Wallis tests, as appropriate. In case of statistically significant results, post hoc testing was performed conform Bonferroni. To investigate differences between colon and rectal surgery in LoS and CCI, statistical methods included Chi-square and Kruskal–Wallis testing for two groups and more than two groups, respectively. A *p* < 0.05 was considered statistically significant.

## Results

In the period January 2020–June 2021, a total of 156 patients were scheduled for elective CRC surgery. The program was declined by 22 patients (14.1%), resulting in 134 patients that started the program. A total of 33 patients (24.6%) did not complete the program, due to either drop out (81.8%) or rescheduled operation (18.2%). A total of 101 patients were included, who performed at least one exercise test at T0 and T1. Baseline characteristics and surgical data are shown in Table 1.

**Table 1:** Baseline characteristics of the study population.

	Total cohort *n* = 101	Colon *n* = 65	Rectum *n* = 36	*p*
**Sex (M:F)**	52:49	30:35	22:14	0.212
**Age (years)**	69.7 ± 12.7	70.1 ± 13.5	69.1 ± 11.3	0.427
<49	6 (5.9)	4 (6.2)	2 (5.6)	
50–60	13 (12.8)	6 (9.2)	7 (19.4)	
61–70	27 (26.7)	16 (24.6)	11 (30.6)	
71–80	36 (35.6)	26 (40)	10 (27.8)	
81>	19 (18.8)	13 (20)	6 (16.7)	
**ASA (%)**				0.876
I	8 (7.9)	5 (7.7)	3 (8.3)	
II	65 (64.4)	43 (66.2)	22 (61.1)	
III	28 (27.7)	17 (26.2)	11 (30.6)	
**Charlson Comorbidity Index**	5.4 (1.8)	5.5 (1.8)	5.3 (1.7)	0.535
**BMI (kg/m^2^)**	27 ± 4.5	27.2 ± 4.5	26.6 ± 4.4	0.521
**Neo adjuvant treatment**	11 (10.9)		11 (30.6)	
Short course			6 (54.5)	
Long course			5 (45.5)	
**Pathological T stadium (%)**				0.328
T0	1 (1)		1 (2.9)	
T1	12 (12.4)	9 (14.5)	3 (8.6)	
T2	26 (26.8)	14 (22.6)	12 (34.3)	
T3	38 (39.2)	24 (38.7)	14 (40)	
T4	20 (20.6)	15 (24.2)	5 (14.3)	
**Pathological N stadium (%)**				0.524
N0	68 (69.4)	43 (69.4)	25 (69.4)	
N1	24 (24.5)	14 (22.6)	10 (27.8)	
N2	6 (6.1)	5 (8.1)	1 (2.8)	
**Pathological M stadium (%)**				0.531
M0	94 (95.9)	59 (95.2)	35 (97.2)	
M1	4 (4.1)	3 (4.8)	1 (2.8)	
**Surgical procedure (%)**				
Right-sided colectomy	41 (40.6)	41 (63.1)		
Transversectomy	1 (1)	1 (2)		
Left-sided colectomy	10 (9.9)	10 (15.4)		
Sigmoid resection	13 (12.9)	13 (20)		
LAR	18 (17.8)		18 (50)	
Rectosigmoid resection	4 (4)		4 (11.1)	
TATME	1 (1)		1 (2.8)	
APR	12(11.9)		12 (33.3)	
**Surgical technique (%)**				0.262
Laparoscopic	83 (82.1)	50 (76.9)	33 (91.7)	
Robot assisted	4 (4)	3 (4.6)	1 (2.8)	
Conversion rate	10 (9.9)	9 (13.8)	1 (2.8)	
Open	4 (4)	3 (4.6)	1 (2.8)	

Value are presented as mean ± SD or as *n* (%).

APR: abdominal perianal resection; ASA: American Society of Anesthesiologists; BMI: body mass index; LAR: low anterior resection; M: metastases status; N: node status; T: tumor status; TATME: transanal total mesorectal excision.

No serious adverse events due to the program were reported. The median number of training sessions scheduled was 9 [7–11], whereas the median number of training sessions attended was 8.5 [7–11]. Subsequently, adherence to planned training sessions in the included population showed a median of 100% [98–100].

### Primary outcome

#### Aerobic fitness

At the SRT, patients improved in absolute WR_peak_ on average by 16.7% (mean ± SD improvement of 28.7 ± 28.4 W; *p* < 0.001) after the prehabilitation program (Table 2), whereas WR_peak_ normalized for body mass also improved with 16.7% (improvement of 0.36 W/kg body mass; *p* < 0.001).

**Table 2:** Physical fitness changes following the multimodal prehabilitation program of patients who completed the performance test(s) before and after the intervention.

	T0	T1	Absolute Δ score^a^	Relative Δ score (%)^a^
**SRT (*n* = 89)**				
WR_peak_ (W)	207 ± 71.2	235.7 ± 77.8	+28.7	+16.7
WR_peak_ (W/kg body mass)	2.7 ± 0.9	3.0 ± 1.0	+0.4	+17.1
**6MWT (*n* = 44)**	522 ± 111	560 ± 117	+37.5	+7.7
**6MWT progression ≥ 20 m**				
Yes			*n* = 25 (56.8%)	
No			*n* = 19 (43.2%)	
**1RM low row (*n* = 65)**				
Load (kg)	46.9 ± 13.4	55.6 ± 14.7	+8.7	+21.2
Load (kg/kg body mass)	0.6 ± 0.1	0.7 ± 0.2	+0.1	+21.8
**1RM chest press (*n* = 77)**				
Load (kg)	29.5 ± 12.8	35.7 ± 15.2	+6.2	+24.8
Load (kg/kg body mass)	0.4 ± 0.1	0.5 ± 0.2	+0.1	+25.2
**1RM leg press (*n* = 79)**				
Load (kg)	112.4 ± 35.2	145.6 ± 45.5	+33.2	+31.9
Load (kg/kg body mass)	1.4 ± 0.4	1.9 ± 0.5	+0.4	+32.5
**1RM lateral pull down (*n* = 78*)***				
Load (kg)	37.3 ± 10	42.9 ± 11.4	+5.6	+16.1
Load (kg/kg body mass)	0.5 ± 0.1	0.6 ± 0.1	+0.1	+16.5

Values are presented as mean **±** SD.

Abbreviations: 1RM: one-repetition maximum; 6MWT: six-minute walk test; SRT: steep ramp test; T0: assessment before the program; T1: assessment after the program; WR_peak_: work rate at peak exercise.

a Difference between T0 and T1 (Δ score) was *p* < 0.001 for all tests.

**Figure 1:**
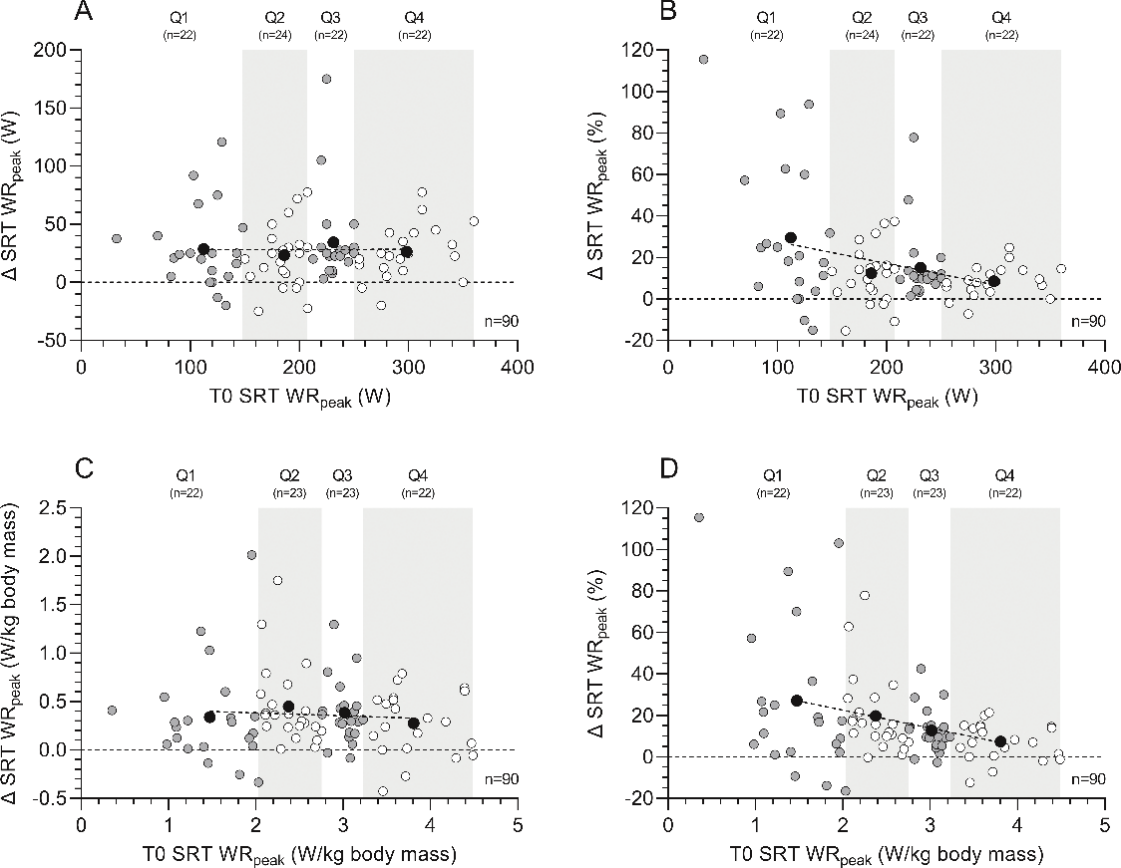
Aerobic fitness changes (Δ, T1 minus T0) following the multimodal prehabilitation program of patients who completed the SRT before and after the intervention: changes in absolute SRT WR_peak_ (W) (graph A), relative changes in SRT WR_peak_ (%) (graph B), changes in absolute SRT WR_peak_ normalized for body mass (W/kg body mass) (graph C), and relative changes in SRT WR_peak_ normalized for body mass (%) (graph D). Based on SRT performance at baseline (T0), patients were divided in quartiles. White and grey dots represent individual patient data within each quartile, whereas black dots represent the mean value within each quartile and the dotted line represents the linear regression line of the black dots to show the effect of aerobic fitness at baseline on the change score. Abbreviations: SRT=steep ramp test; T0=assessment before the program; T1=assessment after the program; WR_peak_=work rate at peak exercise.

Figure 1 presents the change (Δ, T1 minus T0) in SRT performance, in which participants were divided in quartiles based on baseline (T0) SRT performance to indicate the effect of baseline aerobic fitness on the change score. Within the different quartiles, patients demonstrated an increase of 29.5%, 13.2%, 15.0, and 8.2% in absolute WR_peak_ values for Q1, Q2, Q3, and Q4, respectively (Figure 1B, *p* = 0.146), whereas an increase of 27.1%, 19.7%, 12.7%, and 7.4% was observed in WR_peak_ values normalized for body mass for Q1, Q2, Q3, and Q4, respectively (Figure 1D, *p* = 0.049). Dividing the population in age groups, a significant difference was found in ΔWR_peak_ (*p* = 0.016), while post hoc testing showed no statistically significant results. There was no difference in Δ SRT performance comparing males and females (*p* = 0.543), nor when dividing the study population into ASA groups (I, II, III) (*p* = 0.189). When looking at absolute ΔWR_peak_, no significant difference was found when comparing the population on sex (*p* = 0.585), age groups (*p* = 0.256), or ASA groups (*p* = 0.175).

### Secondary outcomes

#### Functional walking capacity

**Figure 2:**
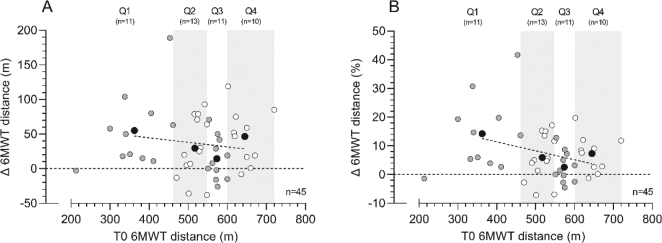
Six-minute walk test (6MWT) distance changes (Δ, T1 minus T0) following the multimodal prehabilitation program of patients who completed the 6MWT before and after the intervention: changes in 6MWT distance (m) (graph A) and relative changes in 6MWT distance (%) (graph B). Based on 6MWT performance at baseline (T0), patients were divided in quartiles. White and grey dots represent individual patient data within each quartile, whereas black dots represent the mean value within each quartile and the dotted line represents the linear regression line of the black dots to show the effect of functional walking distance at baseline on the change score. Abbreviations: 6MWT: 6-minute walk test; T0=assessment before the program; T1=assessment after the program.

Regarding the 6MWT, patients improved on average by 7.3% (+37.5 m, *p* < 0.001) after the prehabilitation program, of which 56.8% of the patients showed a clinically relevant improvement ≥ 20 m (Table 2, Figure 2). The change in 6MWT distance between T0 and T1 showed an increase of 14.2%, 5.9%, 2.6%, and 7.3% for Q1, Q2, Q3, and Q4, respectively (Figure 2B, *p* = 0.027), while post hoc testing showed the largest difference between Q1 and Q3 (*p* = 0.020). No significant differences were found for age, sex, or ASA score.

#### Muscle strength

At the muscle strength tests, patients on average demonstrated an improvement in absolute load varying from 16.1% to 31.9% for the low row, chest press, leg press, and lateral pull down following the prehabilitation program (*p* < 0.001, Table 2). Normalized for body mass, average improvements ranged from 16.5% to 32.5% (all *p* < 0.001).

**Figure 3:**
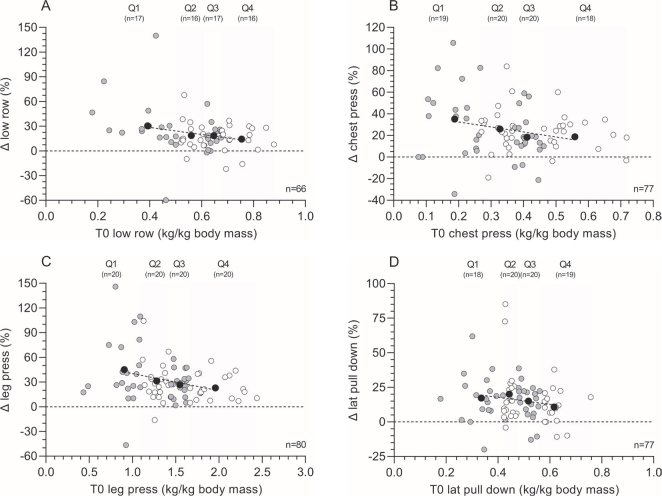
One-repetition maximum (1RM) muscle strength changes (Δ, T1 minus T0) following the multimodal prehabilitation program of patients who completed the tests before and after the intervention: relative changes in low row load expressed in kg normalized for body mass (%) (graph A), relative changes in chest press load expressed in kg normalized for body mass (%) (graph B), relative changes in leg press load expressed in kg normalized for body mass (%) (graph C), and relative changes in lateral pull down load expressed in kg normalized for body mass (%) (graph D). Based on muscle strength at baseline (T0), patients were divided in quartiles. White and grey dots represent individual patient data within each quartile, whereas black dots represent the mean value within each quartile and the dotted line represents the linear regression line of the black dots to show the effect of muscle strength at baseline on the change score. Abbreviations: T0=assessment before the program; T1=assessment after the program.

Within the different quartiles of the absolute 1RM low row load, patients demonstrated an increase of 28.9%, 22.4%, 15.9%, and 12.7% for Q1, Q2, Q3, and Q4, respectively (*p* = 0.029). The relative 1RM low row load, patients increased with 30.3%, 18.6%, 18.8%, and 14.3%, for Q1, Q2, Q3, and Q4, respectively (Figure 3A, *p* = 0.036). No statistically significant differences were found between age groups, ASA score, or sex.

Absolute values for the 1RM chest press load increased with 38.6%, 18.3%, 20.9%, and 21.7% in Q1, Q2, Q3, and Q4, respectively (*p* = 0.004), whereas patients improved with 35.1%, 26%, 18.4%, and 22.5% in relative 1RM chest press load for Q1, Q2, Q3, and Q4, respectively (Figure 3B, *p* = 0.047). Post hoc testing showed the biggest difference between Q1 and Q3 (*p* = 0.044). A significant difference in absolute (*p* = 0.009) and relative (*p* = 0.042) Δ chest press load was found between males and females, with males demonstrating higher change scores. No significant differences were found in either ASA score or age subgroups.

Regarding the absolute 1RM leg press load, patients demonstrated an increase of 45.1%, 23.0%, 28.0%, and 26.6% for Q1, Q2, Q3, and Q4, respectively (*p* = 0.004). The different quartiles of the relative 1RM leg press load showed an increase of 45.1%, 31.2%, 26.8 %, and 22.8% for Q1, Q2, Q3, and Q4, respectively (Figure 3C; *p* = 0.006). Post hoc testing showed a higher absolute Δ leg press in age group 50–60 year versus 70–80 year (*p* = 0.018). No differences were found, when dividing the population in either ASA groups or between males and females.

After analyzing the different quartiles of the absolute 1RM lateral pull down load, patients demonstrated an increase of, respectively, 17.5%, 17.0%, 16.8%, and 10.8% for Q1, Q2, Q3, and Q4 (*p* = 0.867). Different quartiles of the relative 1RM lateral pull down load showed an increase of 18.1%, 19.1%, 15.0%, and 10.8% for Q1, Q2, Q3, and Q4, respectively (Figure 3B, *p* = 0.353). No statistically significant differences were found when dividing the population in ASA or age subgroups, or between sexes.

#### Surgical outcomes

Patients had a median [IQR] LoS of 4.0 [3.0-6.0] days. The postoperative complication rate was 25.7%, with a mean (± SD) CCI score of 6.2 (± 12.1). Median [IQR] CCI was 0.0 [0.0–8.7]. There was no difference in median LoS (4.0 [3.0–6.0] and 4.0 [3.0–7.0] days, respectively, *p* = 0.997) or median CCI (0.0 [0.0–0.0] and 0 [0.0–17.9], respectively, *p* = 0.479) between colon and rectal surgery.

## Discussion

This study assessed preoperative physical fitness changes in patients scheduled for elective CRC surgery participating in a multimodal prehabilitation program that was implemented in the CRC care pathway as standard care, while also evaluating who benefits most. Main findings of this single-center real-world prospective cohort study showed an increase in preoperative physical fitness, assessed with the SRT, 6MWT, and 1RM of different muscle groups. Although the entire cohort improved in physical fitness, relatively the greatest improvements were observed in patients with the lowest physical fitness level at baseline.

RCTs have consistently shown that prehabilitation programs improve physical fitness [7] mostly assessed by performing a CPET. While RCTs provide evidence of efficacy, real-world studies produce evidence of therapeutic effectiveness in real-world practice settings [31]. For truly defining the effectiveness of patient selection in prehabilitation, RCTs are preferable compared to cohort studies. However, due to the increase of evidence for prehabilitation it is considered unethical to withhold patients from prehabilitation. Since the current study results are obtained in real-world practice, results regarding the ability of prehabilitation to improve physical fitness might be better generalizable for the practicing clinician.

This study shows the preoperative physical fitness level of patients scheduled for elective oncological colorectal surgery in a large Dutch teaching hospital. Additionally, our study results indicate that an implemented prehabilitation program enhances preoperative aerobic fitness, functional walking capacity, and muscle strength. All exercise tests results improved significantly. When looking at preoperative physical fitness in recent literature, the 6MWT is most frequently used to assess physical fitness, with mean preoperative values before prehabilitation varying from 325 to 452 m that increased by 11–37 m at the group level after the program [32]. Our study showed similar results with a mean increase of 37.5 m (+7.3%; *p* < 0.001). The (modified) STR has a strong correlation between its primary outcome (WR_peak_) and oxygen uptake at peak exercise at the sophisticated CPET and might therefore be a suitable practical test to use in routine practice for monitoring aerobic fitness [27, 33]. SRT performance normalized for body mass improved by 16.7% (*p* < 0.001) in the this study cohort. Muscle strength normalized for body mass improved on average by 21.8%, +25.2%, +32.5%, and +16.5% for low row, chest press, leg press, and lateral pull down, respectively (all *p* < 0.001). In Supplementary Table 1, baseline preoperative physical fitness values are presented for males and females separately.

To date, it is unknown which group of patients benefits most from prehabilitation. Literature on prehabilitation programs mostly describe changes in physical fitness in high-risk patients [9, 10]. However, the PREHAB trial, the largest study on prehabilitation in CRC surgery, found a moderate but statistically significant improvement of physical fitness without patient selection [11]. In this study, the 25% patients with the lowest baseline exercise test score (Q1) improved the most following the prehabilitation program. A trend toward less progression in physical fitness was observed from group Q2 to Q1, from group Q3 to Q2, and from group Q4 to Q3. The question remains whether exercise prehabilitation supervised by a physical therapist should be reserved for patients who have a baseline score below a certain threshold value, especially since all patients seem to improve more or less equally in absolute values (Figure 1 and 2 and supplementary Figures). Since multimodal prehabilitation seems to have such a positive influence on both surgical outcomes and physical fitness it is receommended to await the results of large population-based studies before we withhold fitter patients these potential benefits.

In this present study patients had an overall complication rate of 27.5%, which is within the range of the most recent Dutch benchmark study [2]. However, this was a single-center study with no control group, in which surgery was predominantly minimally invasive colon surgery; therefore, the implications of the complication rate from this study cannot be compared with recent literature.

## Limitations

One major limitation of this study is the risk of selection bias, as patients who are willing to perform physical exercise training are more susceptible of participating in the program. However, this program was offered to every patient with an indication for elective colorectal surgery. An additional limitation is that not every included patient performed all exercise tests at T0 and T1.

No randomization was performed as prehabilitation was standard of care. Moreover, the number of patients in different subgroups (quartiles, age groups, ASA category) were limited, which should be kept in mind for defining the effect of the secondary analysis. The last limitations are that the results could be influenced by multiple testing and that a learning effect could have occurred in the functional tests that were repeated.

## Interpretation

Multimodal prehabilitation implemented as part of standard of care preoperatively is associated with improved physical fitness in patients scheduled for elective CRC surgery. The largest improvements were found in patients with the lowest baseline physical fitness level. For truly defining which patients benefit most from prehabilitation, large cohort studies are necessary.

## Previous communication to a society or meeting

ESSO 41.

## Supplementary Material

Multimodal prehabilitation in elective oncological colorectal surgery enhances preoperative physical fitness: a single center prospective real-world data analysis

Multimodal prehabilitation in elective oncological colorectal surgery enhances preoperative physical fitness: a single center prospective real-world data analysis

## Data Availability

Upon request reviewer.
