# Supplementary material for: Multimodal prehabilitation in elective oncological colorectal surgery enhances preoperative physical fitness: a single center prospective real-world data analysis
Source: Acta Oncol. 2024 Feb 21;63:20287. doi: 10.2340/1651-226X.2024.20287 (PMC11332481; doi:10.2340/1651-226X.2024.20287)
Supplement: Multimodal prehabilitation in elective oncological colorectal surgery enhances preoperative physical fitness: a single center prospective real-world data analysis [file AO-63-20287-s1.pdf]

Supplementary material has been published as submitted. It has not been copyedited or typeset by Acta Oncologica.

Supplementary Table 1. Physical fitness changes following the multimodal prehabilitation program of males and females who completed the performance test(s) before and after the intervention.

	**Males**	**Females**
**SRT (n=89)**	(n=46)	(n=43)
T0 WR_{peak} (W)	234.4 ± 71.6	177.8 ± 58.6
T0 WR_{peak} (W/kg body mass)	2.9 ± 0.9	2.48 ± 0.85
T1 WR_{peak} (W)	264.3 ± 77.4	205.2 ± 66.5
T1 WR_{peak} (W/kg body mass)	3.2 ± 0.95	2.85 ± 0.91
**6MWT (n=44)**	n=24	n=20
T0 (m)	506 ± 122	541 ± 96
T1 (m)	547 ± 130	574 ± 101
**1RM low row (n=65)**	n=36	n=29
T0 load (kg)	53.5 ± 11.8	38.7 ± 10.5
T0 load (kg/kg body mass)	0.64 ± 0.1	0.52 ± 0.1
T1 load (kg)	62.4 ± 13.3	47.1 ± 11.8
T1 load (kg/kg body mass)	0.75 ± 0.2	0.64 ± 0.2
**1RM chest press (n=76)**	n=40	n=36
T0 load (kg)	35.6 ± 12.3	21.9 ± 9.1
T0 load (kg/kg body mass)	0.44 ± 0.1	0.30 ± 0.1
T1 load (kg)	43.5 ± 13.9	27.2 ± 11.6
T1 load (kg/kg body mass)	0.54 ± 0.2	0.37 ± 0.1
**1RM leg press (n=79)**	n=43	n=36
T0 load (kg)	123.6 ± 34.2	99 ± 31.9
T0 load (kg/kg body mass)	1.5 ± 0.4	1.4 ± 0.4
T1 load (kg)	158 ± 47.5	130.7 ± 38.4
T1 load (kg/kg body mass)	1.9 ± 0.5	1.8 ± 0.5
**1RM lateral pull down (n=78)**	n=43	n=35
T0 load (kg)	42.5 ± 8.9	30.8 ± 7.2
T0 load (kg/kg body mass)	0.5 ± 0.1	0.4 ± 0.1
T1 load (kg)	48.8 ± 9.6	35.7 ± 9.1
T1 load (kg/kg body mass)	0.6 ± 0.1	0.5 ± 0.1

Values are presented as mean ± SD
Abbreviations: 1RM=one-repetition maximum; 6MWT=six-minute walk test; SRT=steep ramp test; T0=assessment before the program; T1=assessment after the program; WR_{peak}=work rate at peak exercise.
