# Supplementary material for: Multimodal prehabilitation in elective oncological colorectal surgery enhances preoperative physical fitness: a single center prospective real-world data analysis
Source: Acta Oncol. 2024 Feb 21;63:20287. doi: 10.2340/1651-226X.2024.20287 (PMC11332481; doi:10.2340/1651-226X.2024.20287)
Supplement: Multimodal prehabilitation in elective oncological colorectal surgery enhances preoperative physical fitness: a single center prospective real-world data analysis [file AO-63-20287-s2.pdf]

Supplementary material has been published as submitted. It has not been copyedited or typeset by Acta Oncologica.

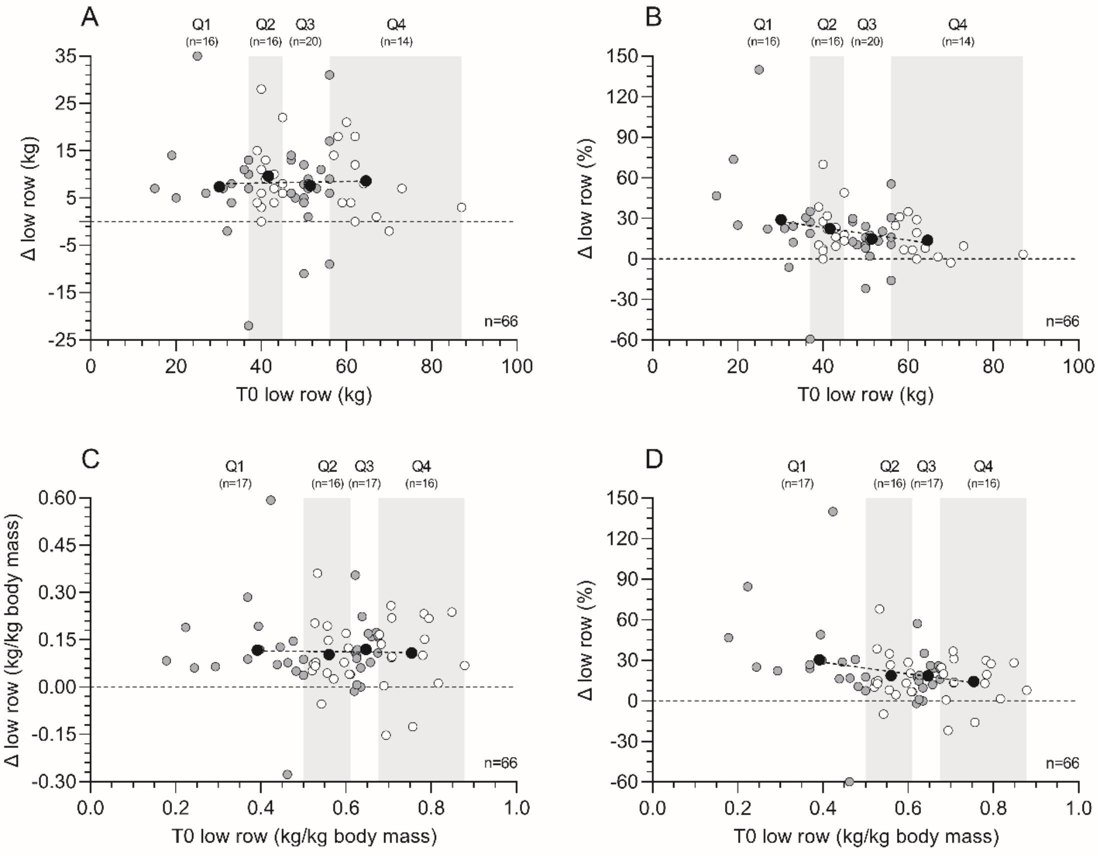

Supplementary Figure 1. 1RM muscle strength changes (Δ, T1 minus T0) following the multimodal prehabilitation program of patients who completed the low row before and after the intervention: changes in absolute low row load (kg) (graph A), relative changes in absolute low row load (%) (graph B), changes in low row load normalized for body mass (kg/kg body mass) (graph C), and relative changes in low row load normalized for body mass (%) (graph D). Based on low row performance at baseline (T0), patients were divided in quartiles. White and grey dots represent individual patient data within each quartile, whereas black dots represent the mean value within each quartile and the dotted line represents the linear regression line of the black dots to show the effect of low row performance at baseline on the change score. Abbreviations: T0=assessment before the program; T1=assessment after the program.

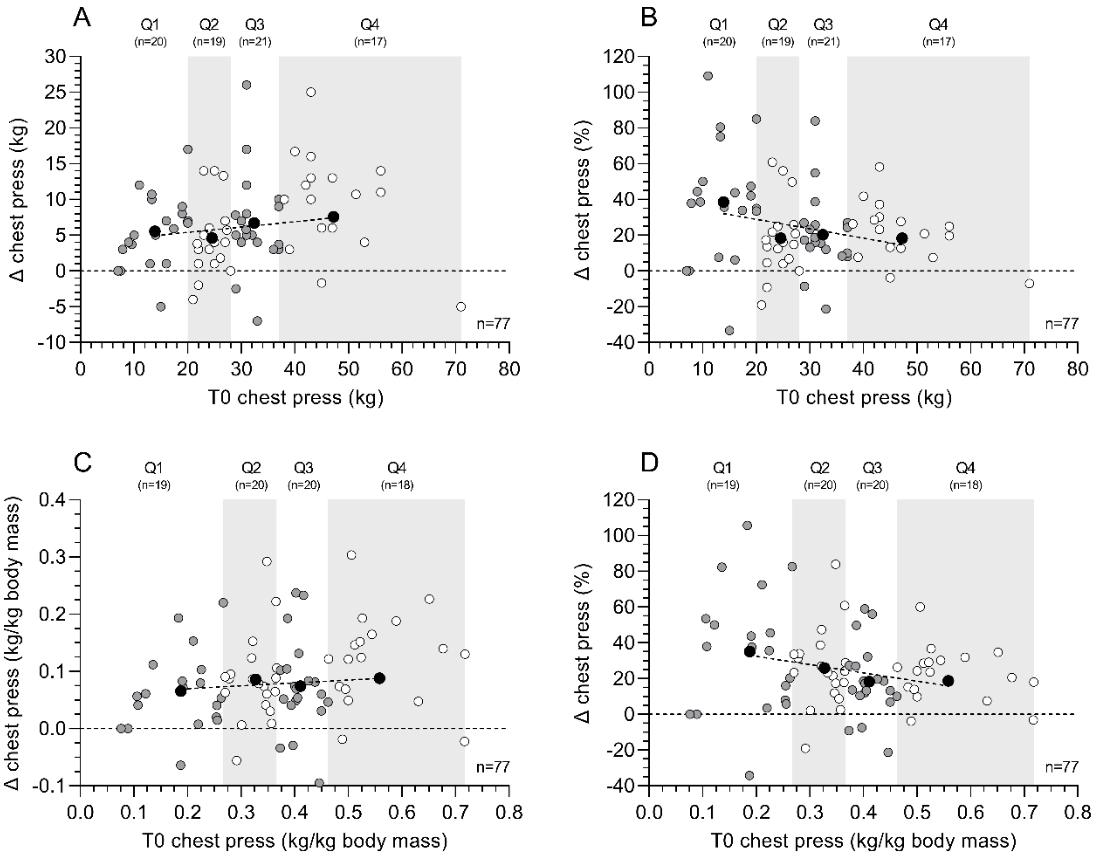

Supplementary Figure 2. 1RM muscle strength changes (Δ, T1 minus T0) following the multimodal prehabilitation program of patients who completed the chest press before and after the intervention: changes in absolute chest press load (kg) (graph A), relative changes in absolute chest press load (%) (graph B), changes in chest press load normalized for body mass (kg/kg body mass) (graph C), and relative changes in chest press load normalized for body mass (%) (graph D). Based on chest press performance at baseline (T0), patients were divided in quartiles. White and grey dots represent individual patient data within each quartile, whereas black dots represent the mean value within each quartile and the dotted line represents the linear regression line of the black dots to show the effect of chest press performance at baseline on the change score. Abbreviations: T0=assessment before the program; T1=assessment after the program.

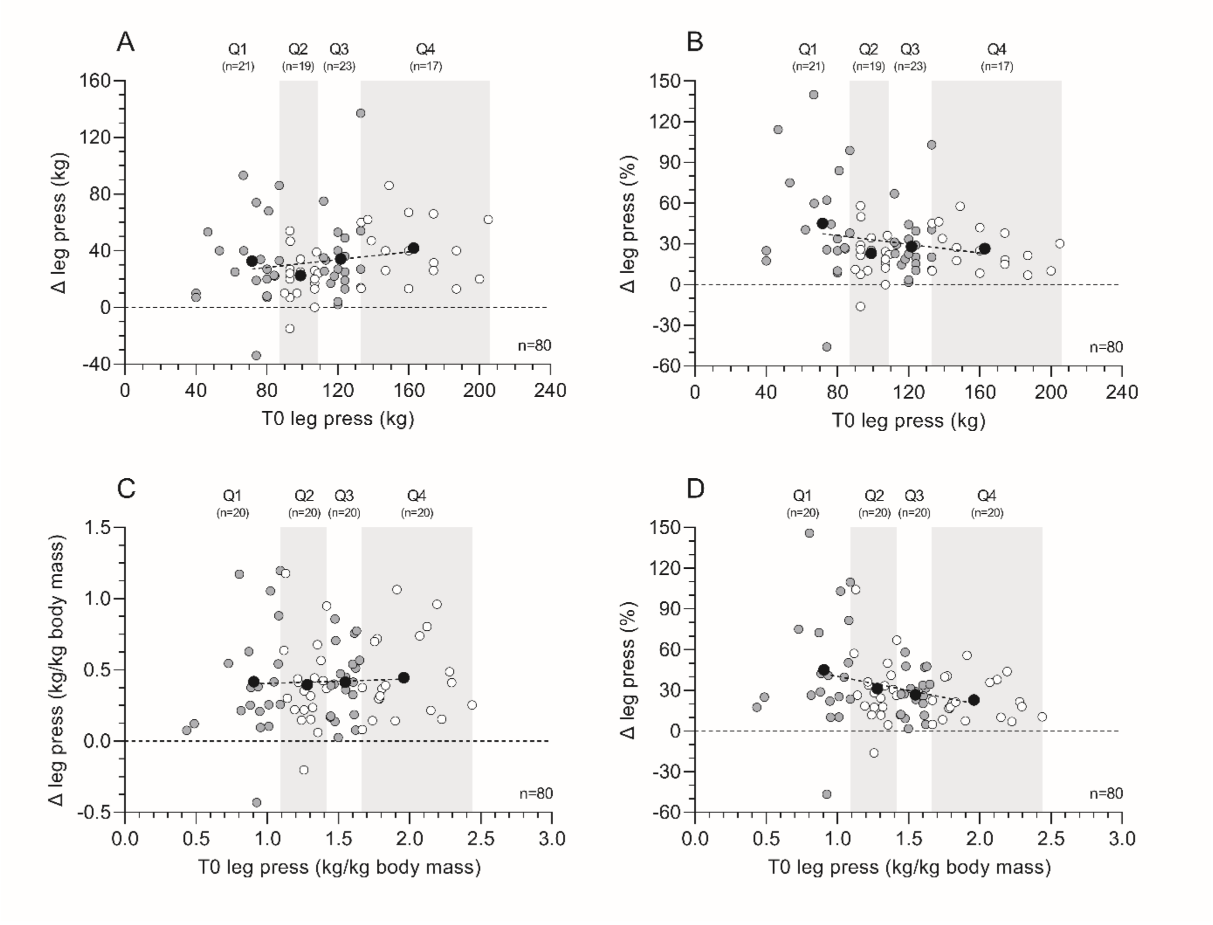

Supplementary Figure 3. 1RM muscle strength changes (Δ, T1 minus T0) following the multimodal prehabilitation program of patients who completed the leg press before and after the intervention: changes in absolute leg press load (kg) (graph A), relative changes in absolute leg press load (%) (graph B), changes in leg press load normalized for body mass (kg/kg body mass) (graph C), and relative changes in leg press load normalized for body mass (%) (graph D). Based on leg press performance at baseline (T0), patients were divided in quartiles. White and grey dots represent individual patient data within each quartile, whereas black dots represent the mean value within each quartile and the dotted line represents the linear regression line of the black dots to show the effect of leg press performance at baseline on the change score. Abbreviations: T0=assessment before the program; T1=assessment after the program.

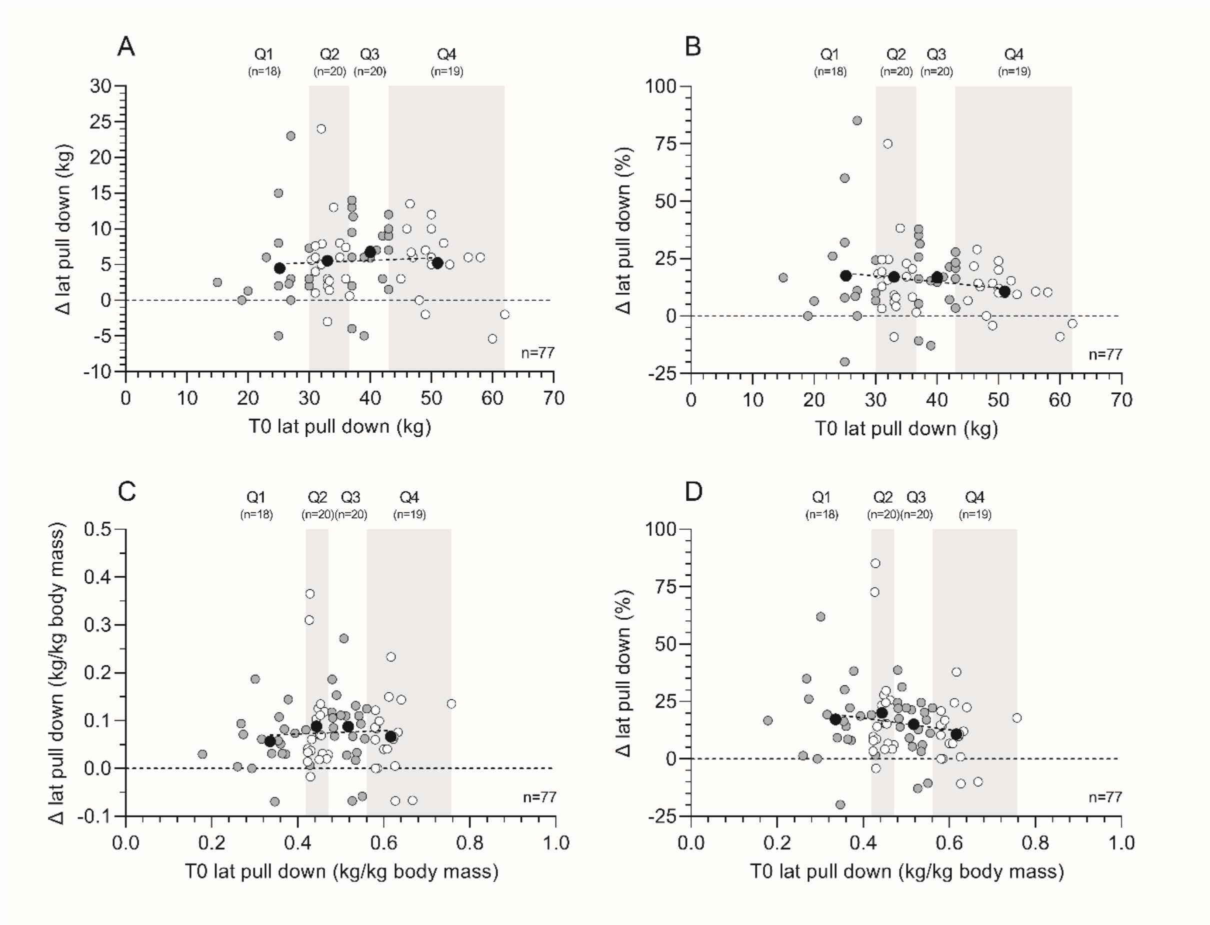

Supplementary Figure 4. 1RM muscle strength changes (Δ, T1 minus T0) following the multimodal prehabilitation program of patients who completed the lateral pull down before and after the intervention: changes in absolute lateral pull down load (kg) (graph A), relative changes in absolute lateral pull down load (%) (graph B), changes in lateral pull down load normalized for body mass (kg/kg body mass) (graph C), and relative changes in lateral pull down load normalized for body mass (%) (graph D). Based on lateral pull down performance at baseline (T0), patients were divided in quartiles. White and grey dots represent individual patient data within each quartile, whereas black dots represent the mean value within each quartile and the dotted line represents the linear regression line of the black dots to show the effect of lateral pull down performance at baseline on the change score. Abbreviations: T0=assessment before the program; T1=assessment after the program.

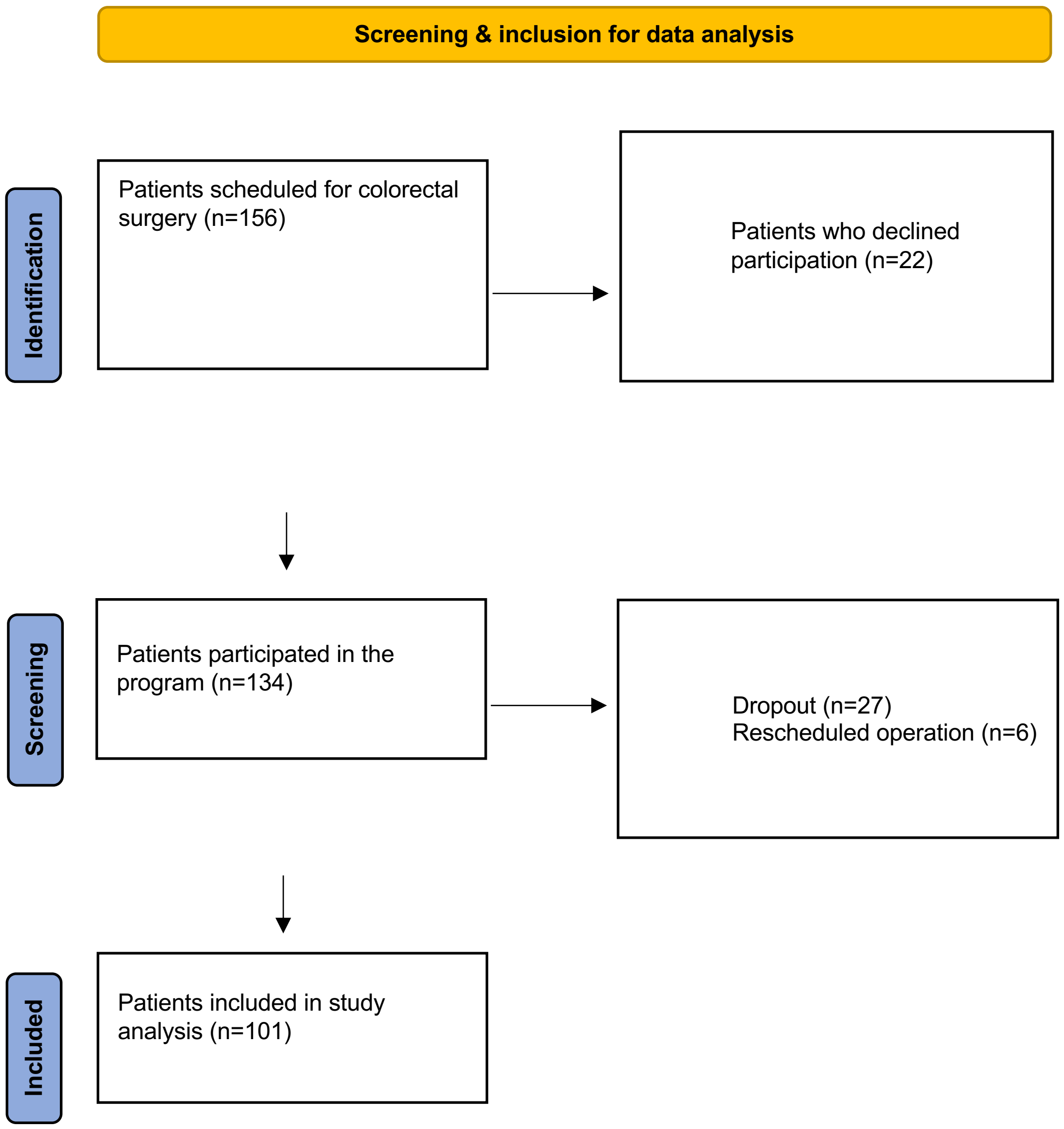
Screening & inclusion for data analysis
Identification
Patients scheduled for colorectal surgery (n=156)
Patients who declined participation (n=22)
Screening
Patients participated in the program (n=134)
Dropout (n=27)
Rescheduled operation (n=6)
Included
Patients included in study analysis (n=101)
